# Ontogeny of Synovial Macrophages and the Roles of Synovial Macrophages From Different Origins in Arthritis

**DOI:** 10.3389/fimmu.2019.01146

**Published:** 2019-05-24

**Authors:** Jiajie Tu, Wenming Hong, Yawei Guo, Pengying Zhang, Yilong Fang, Xinming Wang, Xiaoyun Chen, Shanshan Lu, Wei Wei

**Affiliations:** ^1^Key Laboratory of Anti-Inflammatory and Immune Medicine, Ministry of Education, Anhui Collaborative Innovation Center of Anti-Inflammatory and Immune Medicine, Institute of Clinical Pharmacology, Anhui Medical University, Hefei, China; ^2^Department of Neurosurgery, First Affiliated Hospital of Anhui Medical University, Hefei, China

**Keywords:** SM, ontogeny, ESM, BMSM, CIA, RA

## Abstract

The ontogeny of macrophages in most organ/tissues in human body has been proven. Due to the limited number and inaccessibility of synovial macrophages (SM), the origin of SM has not been fully illuminated. The objective of this study was designed to investigate the ontogeny of SM and to evaluate the role of SM from different origins in arthritis. Two origins of SM, embryonic SM (ESM) and bone marrow SM (BMSM) were identified in Cx3cr1-EGFP mice, CCR2^−/−^ mice and bone marrow (BM) chimera model by using a stringent sorting strategy. The cellular features, including dynamic total cell number, *in situ* proliferation, phagocytosis and expressions of pro-inflammatory and anti-inflammatory genes, of ESM and BMSM were compared. In addition, ESM and BMSM showed different expression patterns in Rheumatoid Arthritis (RA) patients' synovium and during the developmental process of collagen-induced arthritis (CIA) mice. Taken together, these results demonstrated that the SM at least has two origins, ESM and BMSM. The different cellular property and dynamic expression patterns in RA patients/CIA mice highlight the notion that ESM and BMSM might play different role in arthritis.

## Introduction

In the immune system of human body, macrophages are the first line of defense against exogenous impairment. In traditional opinion, macrophages are differentiated from circulating monocytes. However, a series of recent publications have been shown that the origins of macrophages in different tissues/organs are not exclusively derived from circulating monocytes. Yolk sac and fetal liver are two main origins of macrophages at embryonic stage (1–8). Although macrophages in synovium (synovial macrophages, SM) were identified long time ago (9), the specific ontogeny of SM is still not systematically investigated. In addition, the specific function of SM from different origins has not been clarified in RA patients or animal model of arthritis.

Here we used combinational methods of immunostaining, flow cytometry, and chimera model to explore the SM ontogeny from prenatal, perinatal, neonatal until adulthood stage in mice. Embryonic SM (ESM) was present in mice joint synovium at prenatal stage, bone marrow-derived SM (BMSM) appeared around perinatal stage and these two macrophage populations mixed after birth. The cellular features of ESM and BMSM are quite different, and the expression patterns of ESM and BMSM show a dynamic change during the developmental process of mouse arthritis model, which is further validated by using synovium from RA patients. These results highlighted the notion that the SM that from different origins may play different role in RA, presenting potential pathogenically mechanism and therapeutically targets of RA.

## Materials and Methods

### Mice

This study was approved by experimental animal ethics committee of Anhui Medical University (No. LLSC20160121) (Supplementary Information). C57BL/6 mice (Jackson lab code:000664), Cx3cr1+/GFP mice (Jackson lab code:005582), B6.SJL-Ptprc Pepc /BoyJ mice (CD45.1, Jackson lab code:002014), and CCR2^−/−^ mice (Jackson lab code:004999) were purchased from Jackson lab (US) maintained under pathogen–free conditions at the Animal Experimental Center at the Anhui Medical University, China. All mice were used between E12.5 and 9 weeks, unless stated otherwise. All experiments were carried out according to agreement with protocols approved by the Anhui Medical University Institutional Animal Care and Use Committee. To remove the mice synovium, sacrifice mice by cervical dislocation, remove the knee joint by scissors, and tweezer without breaking femur (to exclude the contamination from bone marrow), wash in PBS (1 min), 75% ethanol (30s), and PBS (1 min). Then expose synovium by cutting off ligament and remove the synovium by tweezer. Removed mice synovium were washed and miced in 1,640 medium (Life Technologies) with penicillin/streptomycin. Dissected mice synovium were digested in 1,640 media containing 1 mg/ml of collagenase type 4 (Sigma) and 0.1 mg/ml of deoxyribonuclease I (Sigma), incubation for 1 h at 37°C (200 rpm). After filtration by using sterile nylonmesh (70 μm, Falcon), the single cells from mice synovium were centrifuged and the cell pellet resuspended in fresh medium for the following sorting: CD45^+^Ly6G^−^CD11c^−^CD169^−^Ly6C^−^F4/80^+^CD11b^+^. Whole experiment carried out under a sterile condition.

### Generation of Bone Marrow Chimeras

8-week-old B6.SJL-Ptprc Pepc /BoyJ mice (CD45.1^+^) mice were lethally irradiated (5 Gy) and then were reconstituted immediately by intravenous infusion of 5 × 10^6^ BM cells from C57BL/6 mice (CD45.2^+^). Mice were maintained for 8 weeks before blood samples and synovium tissues were used for FACS detection of chimeras.

### Patients

This part was approved by the biomedical ethics committee of Anhui Medical University (No. 20160094) (Supplementary Information). Informed consent was obtained from all patients and/or their legal guardians. 20 joint synovium biopsies from osteoarthritis (OA) and RA patients were used in this experiment, respectively. The patients' information was included in the Supplementary Information. The method used for the isolation of SM from synovial tissue was modified from a method previously described (10). Synovium was replaced from joints of patients with OA and RA. Replaced hyperplastic synovium were washed and miced in 1,640 medium (Life Technologies) with penicillin/streptomycin. Dissected synovial tissue were digested in 1,640 media containing 1 mg/ml of collagenase type 4 (Sigma) and 0.1 mg/ml of deoxyribonuclease I (Sigma), incubation for 2 h at 37°C (200 rpm). Tissues were then vortexed and resuspended in fresh media. After filtration by using sterile nylonmesh (70 μm, Falcon), the single cells from RA patients' symovium were centrifuged and the cell pellet resuspended in fresh media for the following sorting: CD45^+^CD15^−^CD1c^−^CD14^+^EMR1(F4/80)^+^CD11b^+^. Whole experiment carried out under a sterile condition.

### Collagen-Induced Arthritis (CIA) Model

CIA Induction and Treatment Type II collagen was dissolved in 0.1 Macetic acid and emulsified with an equal volume of complete Freund's adjuvant to produce a final concentration of 2 mg/ml before incubating overnight at 4°C. DBA/1 mice (Model Animal Research Center of Nanjing University, China) were injected twice with 0.1 ml of this emulsion at the base of the tail. The day of the first immunization was defined as day 0, and the second injection was administered into the back on day 21. Mice were divided into normal group and CIA model group (*n* = 8 per group). The normal and CIA mice were given an equal volume of vehicle.

### H&E Staining, Immunofluorescence and Immunohistrochemistry

The H&E staining, immunofluorescence (IF) and immunohistochemistry (IHC) were carried out according to standard protocol from previous studies (11). All antibodies for IF and IHC experiments are listed in Supplementary Information.

### Flow Cytometry

The flow cytomery was performed according to standard protocol from previous studies (11) by using FACSArial II (BD). Antibodies for flow cytometry are listed in Supplementary Information.

### Proliferation

For the detection of Ki67 expression, 1 × 10^5^ SM were fixed and stained for 60 min at 20°C with anti-Ki67 (Miltenyi), then analyzed by flow cytometer.

### Phagocytosis

Isolated ESM and BMSM (1 × 10^5^ cells) were cultured with Dextran-FITC according to the manufacturer's guidelines (Life Technology) and were analyzed by flow cytometer.

### Real Time-Quantitative Polymerase Chain Reaction (RT-qPCR)

ESM and BMSM were isolated by flow cytometry from the synovium, and then total RNA was extracted from SM with Trizol reagent (Life technology). RNA as reverse-transcribed to cDNA with the PrimeScriptTM RT reagent kit (Takara), and gene expression was assayed by quantitative RT-PCR with SYBR qPCR master mix (Life technology) and the 7,500 qPCR system (Applied Biosystems). cDNA samples were assayed in triplicate and expression was normalized by using endogenous control GAPDH. All primer for qRT-PCR were listed in the Supplementary Information.

### Statistical Analysis

Groups were compared with Student's *t*-test or, for multiple-group comparisons, one-way analysis of variance followed by a Bonferroni post-test with Prism Software (GraphPad Software).

## Results

### The Ontogeny of Synovial Macrophages (SM)

**Figure 1:**
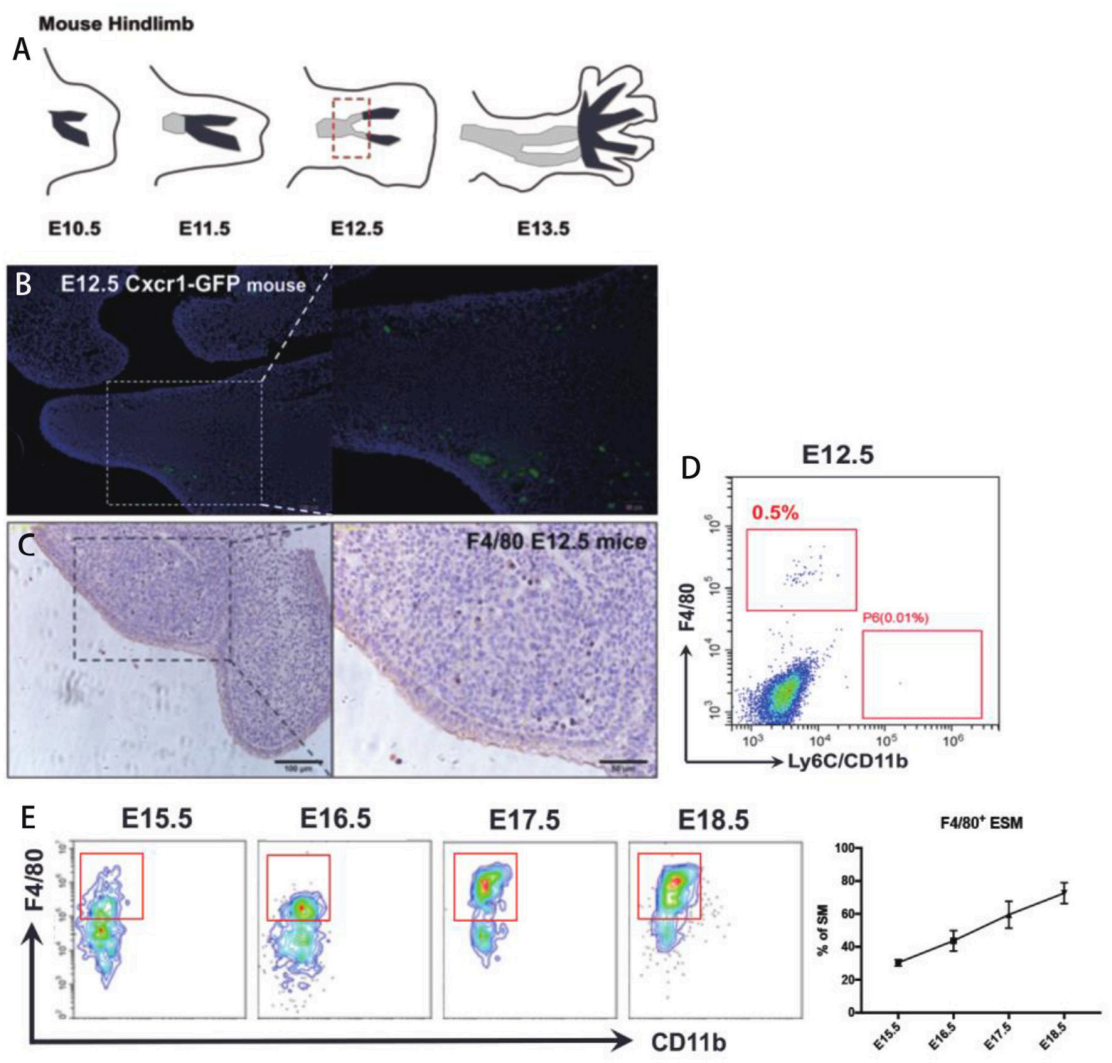
The origin of SM at embryonic stage. **(A)** The development of mice joint synovium during embryonic stage; **(B)** Cx3cr1+/GFP cells localize around joint at E12.5; **(C,D)** Resident macrophage marker F4/80 appears at joint synovium as early as E12.5; **(E)** The number of F4/80^+^ ESM gradually increase with embryonic development. Each bar in the figure represents mean ± SEM of triplicates. 10 mouse embryos were used for each FACS experiment.

A series of previous reports suggested that the macrophages markers F4/80 and CD11b could be used to distinguish embryonic-resident and bone marrow-derived macrophages (1–5, 7). Therefore, we assessed whether expression of F4/80 and CD11b could be used to identify specific macrophage groups in the synovium by using a strict gating strategy CD45^+^Ly6G^−^CD11c^−^CD169^−^F4/80^+^CD11b^+^ (Supplementary Figure 1) (1). It's known that mice joint synovium formed around E12.5 (12, 13) (Figure 1A) We firstly tried to identify the F4/80^+^ and CD11b^+^ SM in mice joint at E12.5. Cx3cr1^+^/GFP mice were also used for SM identification, in which one allele of the gene encoding the chemokine receptor (CX3CR1) is replaced by green fluorescent protein (GFP). Fluorescence results demonstrated that Cx3cr1^+^/GFP cells localized around joint at E12.5 (Figure 1B). In addition, resident macrophage marker F4/80 were used to validate that embryonic-resident SM (ESM) appears as early as E12.5 by using IHC and FACS assays (Figures 1C,D). However, because joint synovium is such a small piece of tissue that only includes several layers of cells, the morphology of synovium is too vague to distinguish from other issues at E12.5. Therefore, we couldn't exclude the possibility that these Cx3cr1^+^/GFP cells or F4/80^+^ ESM localize in other issues around joint while not synovium. With developmental process, the morphology of synovium is quite clear at E15.5 and synovium developed as an intact and specific tissue at P7 (Supplementary Figure 2). The number of F4/80^+^ ESM gradually increased with embryonic development (Figure 1E). However, before E18.5, CD11b^+^ SM couldn't be detected.

**Figure 2:**
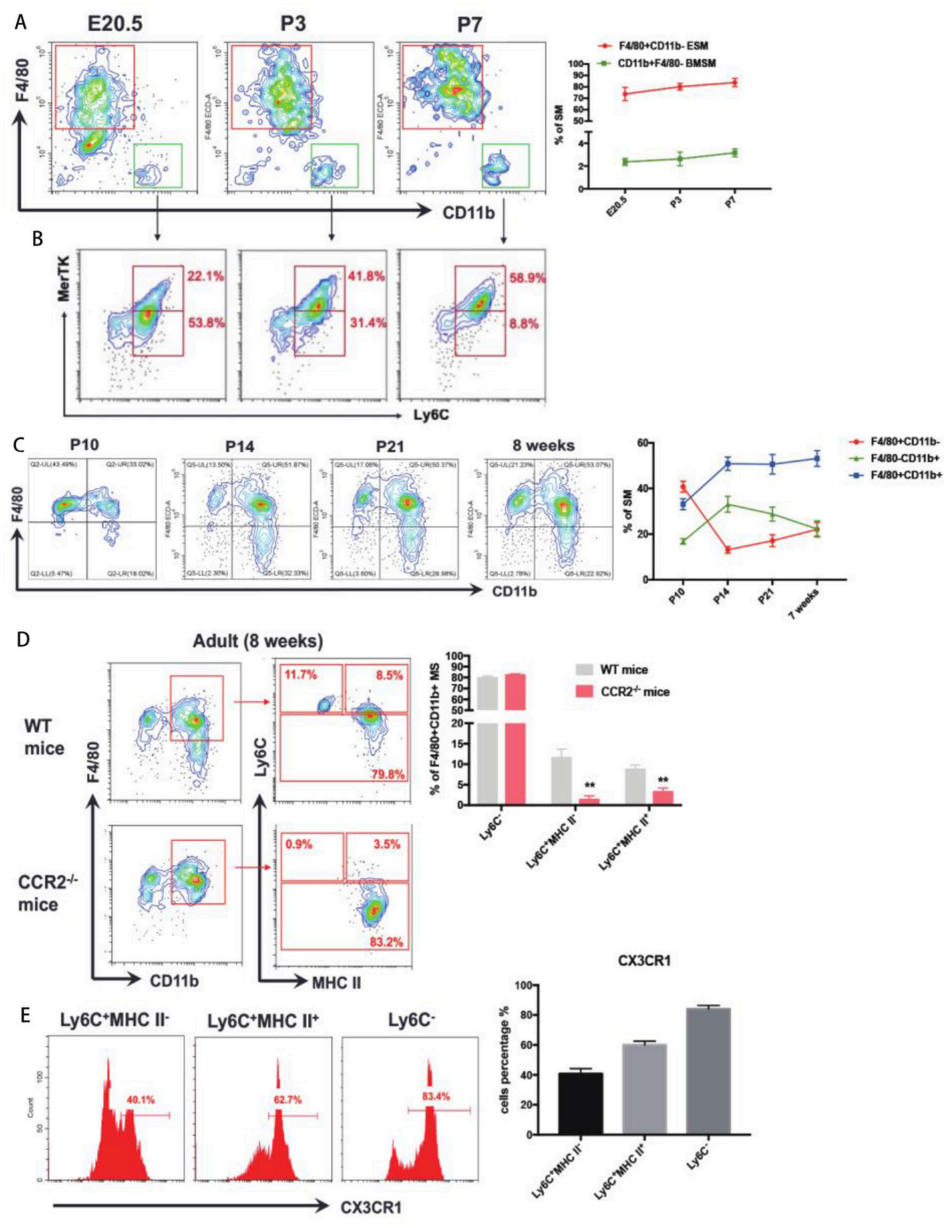
The ontology of SM at perinatal to adult stage. **(A)** F4/80^−^CD11b^+^ BMSM appear in joint synovium at E20.5; **(B)** The expression of Ly6C and MerTK in F4/80^−^CD11b^+^ BMSM; **(C)** ESM gradually increase from neonatal to adult stage and BMSM show the opposite expression pattern. F4/80^+^CD11b^+^ SM appear at neonatal stage and progressively increase up to adult; **(D)** To further divide F4/80^+^CD11b^+^ SM by Ly6C and MHC II in WT mice and CCR2-/- mice. **(E)** The CX3CR1 expression in three subgroups of F4/80^+^CD11b^+^ SM. Each bar in the figure represents mean ± SEM of triplicates. For embryonic stage, 8–10 mouse embryos were used for each FACs experiment. For neonatal stage, 6 cubs were used for each FACS experiment. **P < 0.01.

Around the perinatal period (From E20.5 to P7), after excluding CD11c^+^ dendritic cells (DCs), Siglec-F^+^ eosinophils, and Ly6G^+^ neutrophils, a distinct population of F4/80^−^CD11b^+^ cells were found at E20.5 (Figure 2A). Most CD11b^+^F4/80^−^ BMSM were Ly6C^+^ (Figure 2B), validating that BMSM is indeed derived from monocytes. To distinguish BMSM from monocytes, two pan-macrophage markers, MerTK and CD68, were also evaluated here (Figure 2B and Supplementary Figure 3). The results showed that there is a transition of Ly6C^+^ population to MerTK^+^ BMSM (Figure 2B). The mice bone marrow initially formed around E19 (14), suggesting that the bone marrow-derived synovial macrophages (BMSM) already implant in mice synovium at E20.5. In synovium of both neonatal mice and adult mice, we validated the ESM and BMSM on the expression of the resident-macrophage marker F4/80 and bone marrow-derived macrophage marker CD11b (Figure 2C). In general, F4/80^+^CD11b^−^ ESM gradually increased from neonatal to adult stage and F4/80^−^CD11b^+^ BMSM showed the opposite expression pattern. Interestingly, with the development, there is another mixed population (F4/80^+^CD11b^+^) appears at neonatal stage and progressively increased up to adult (Figure 2C), implying that the distinction between ESM (F4/80^+^CD11b^−^) and BMSM (F4/80^−^CD11b^+^) is becoming more indistinct with the mice development after birth. The numbers of total SM from different origins were calculated accordingly (Supplementary Figure 4).

The F4/80^+^CD11b^+^ SM are the dominant populations in adult mice. To elucidate the origin of this population, we firstly identified the subgroups of F4/80^+^CD11b^+^ SM by using monocyte marker Ly6C and Major histocompatibility complex class II (MHC II, a marker of activated macrophages) in adult wild-type mice. There are three subgroups of F4/80^+^CD11b^+^ SM:Ly6C^−^, Ly6C^+^MHCII^−^, and Ly6C^+^MHCII^+^ (Figure 2D). The Ly6C^+^MHCII^−^ subgroup showed intermediate expression of CX3CR1^int^, which is an established status of circulating monocytes. Ly6C^−^ subgroup expressed high level of CX3CR1^hi^, which often refers to mature resident macrophages. The expression of CX3CR1 in Ly6C^+^MHCII^+^ subgroup is between Ly6C^+^MHCII^−^ and Ly6C^−^ subgroups (Figure 2E).

CCR2^−/−^ mice (Ly6C^+^ monocyte is defective) is used to evaluate the role of classic circulating monocytes to Ly6C^+^ SM. Compared to adult wild-type mice, the Ly6C^+^ SM dramatically decreased in CCR2^−/−^ mice (Figure 2D). However, the percentage of Ly6C^−^ SM from CCR2^−/−^ mice is similar to wild-type mice (Figure 2D), suggesting that CCR2 deficiency didn't affect resident ESM.

### Characterization of SM From Different Origins

**Figure 3:**
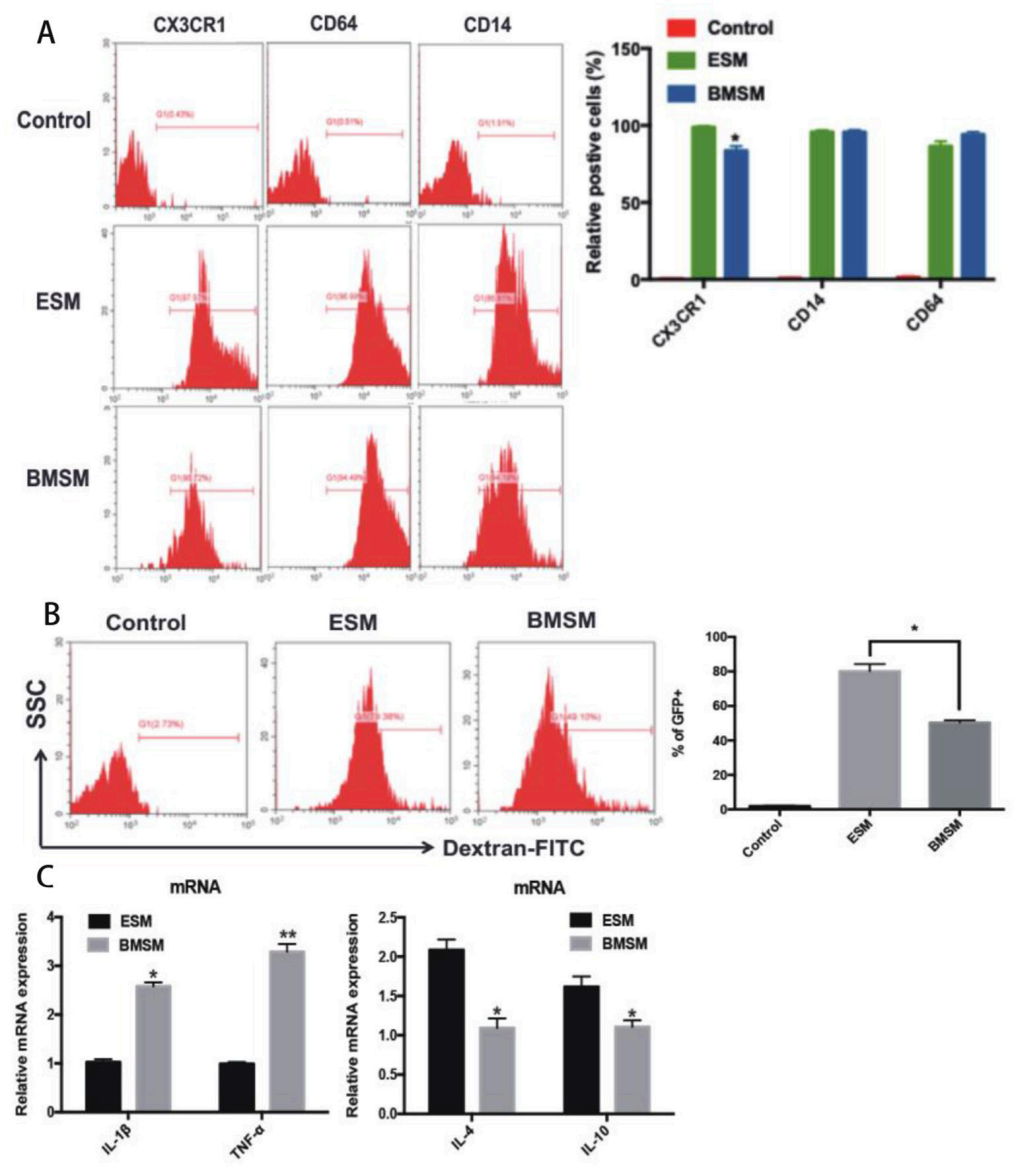
Identification of SM from different origins. **(A)** The expression of the macrophage-specific markers CD64, CD14, and CX3CR1 in ESM and BMSM; **(B)** The comparison of phagocytic ability of ESM and BMSM; **(C)** Expression of IL-1β, TNF-α, IL-4, and IL-10 in ESM and BMSM. Each bar in the figure represents mean ± SEM of triplicates. *P < 0.05, **P < 0.01.

To identify ESM and BMSM, we firstly validated them as macrophages by detecting macrophage-specific markers CD64, CD14, and CX3CR1 (Figure 3A). To further verify the macrophage character, both ESM and BMSM could engulf FITC-labeled Dextran, although ESM showed higher phagocytic activity (Figure 3B). In addition, the expression of some classic pro-inflammatory genes IL-1β and tumor-necrosis factor (TNF)-α and anti-inflammatory genes IL-4 and IL-10 were detected in ESM and BMSM. ESM showed higher expression of IL-4 and IL-10, while BMSM have larger amount of IL-1β and TNF-α (Figure 3C). Therefore, ESM and BMSM demonstrated different phenotypic and functional properties.

**Figure 4:**
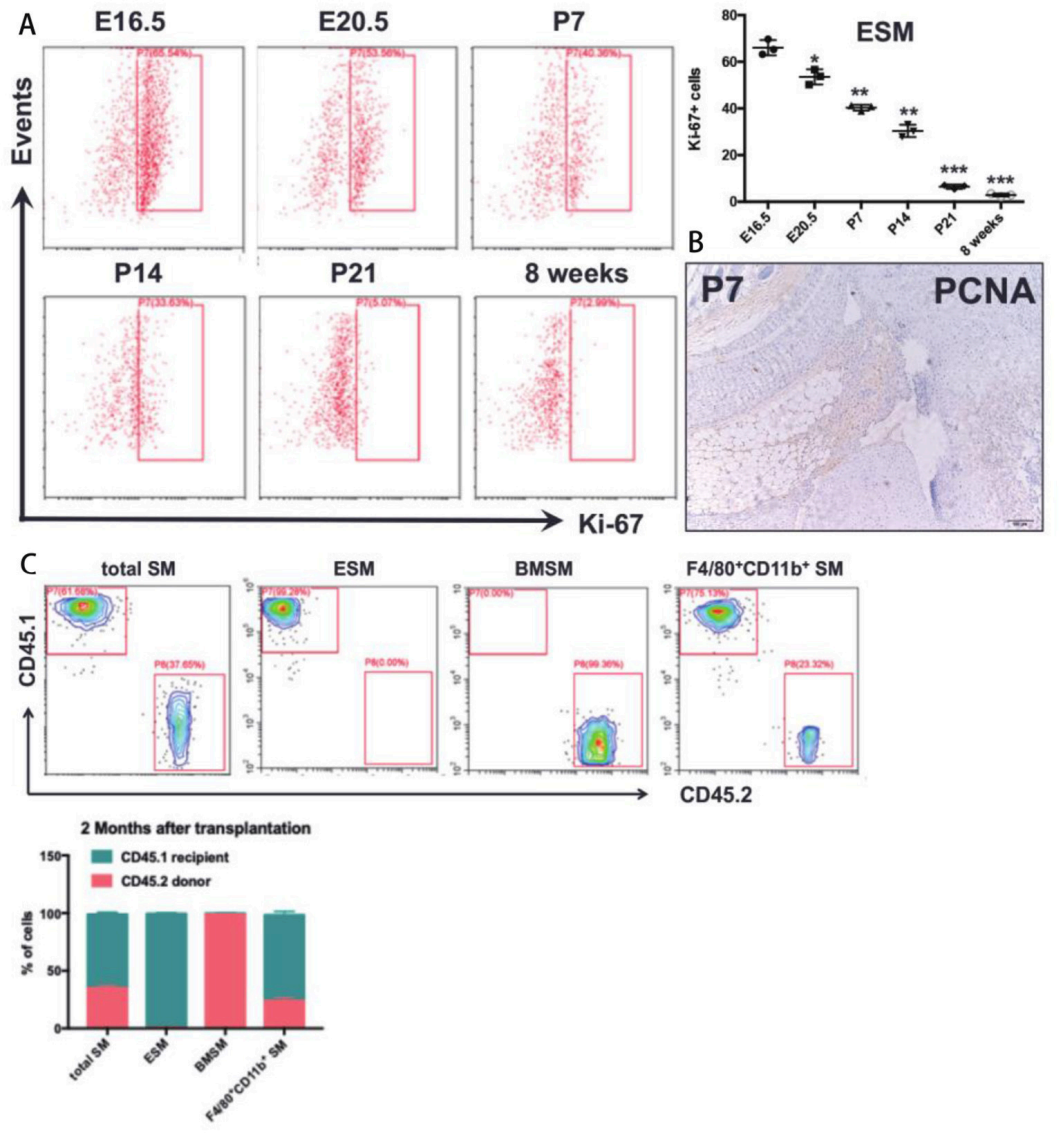
The proliferative ability of SM. **(A)** *in situ* proliferation of the ESM at prenatal and postnatal stages; **(B)** PCNA staining in joint synovium *in situ* at postnatal stage; **(C)** A chimera approach by joining congenic wild-type CD45.1^+^ and CD45.2^+^ mice to assess the contribution of non-host cells in synovium. Each bar in the figure represents mean ± SEM of triplicates. *P < 0.05, **P < 0.01, ***P < 0.001.

We next tested the *in situ* proliferation of ESM and BMSM at prenatal and postnatal stages. In E16.5 mice, around 65% of mature ESM had high Ki67 (a marker that exclusively expressed in proliferating cells), and the Ki67^+^ ESM gradually decreased from prenatal to postnatal stages (Figure 4A). PCNA staining further validated that SM proliferates *in situ* at postnatal stage (Figure 4B). By 8 weeks of age, nearly no Ki67^+^ ESM could be detected (Figure 4A). However, the Ki67^+^ BMSM couldn't be detected neither during prenatal nor postnatal stages (data not shown). Thus, ESM significantly proliferated *in situ* from embryonic stage to neonatal stage, but gradually diminished after birth.

Previous results demonstrated that the SM was derived from both embryonic and bone marrow-derived macrophages. To further reveal the contribution of bone marrow (BM) cells to ESM and BMSM, bone marrow chimera model was established to further elucidate the contribution of ESM and BMSM in total SM. CD45.1 host mice were irradiated and then CD45.2 BM cells was isolated and transplanted by intravenous injection at caudal vein. After 2 months, joint synovium from chimera mice were isolated and analyzed by FACS. For total SM, over 30% cells are from donor. Almost all ESM were recipient origin (Figure 4C), suggesting that ESM was radio-resistant and didn't need a contribution from BM-derived cells. As expected, BMSM was mostly replaced by circulating cells after transplantation (Figure 4C).

### The Role of SM From Different Origins in Arthritis

**Figure 5:**
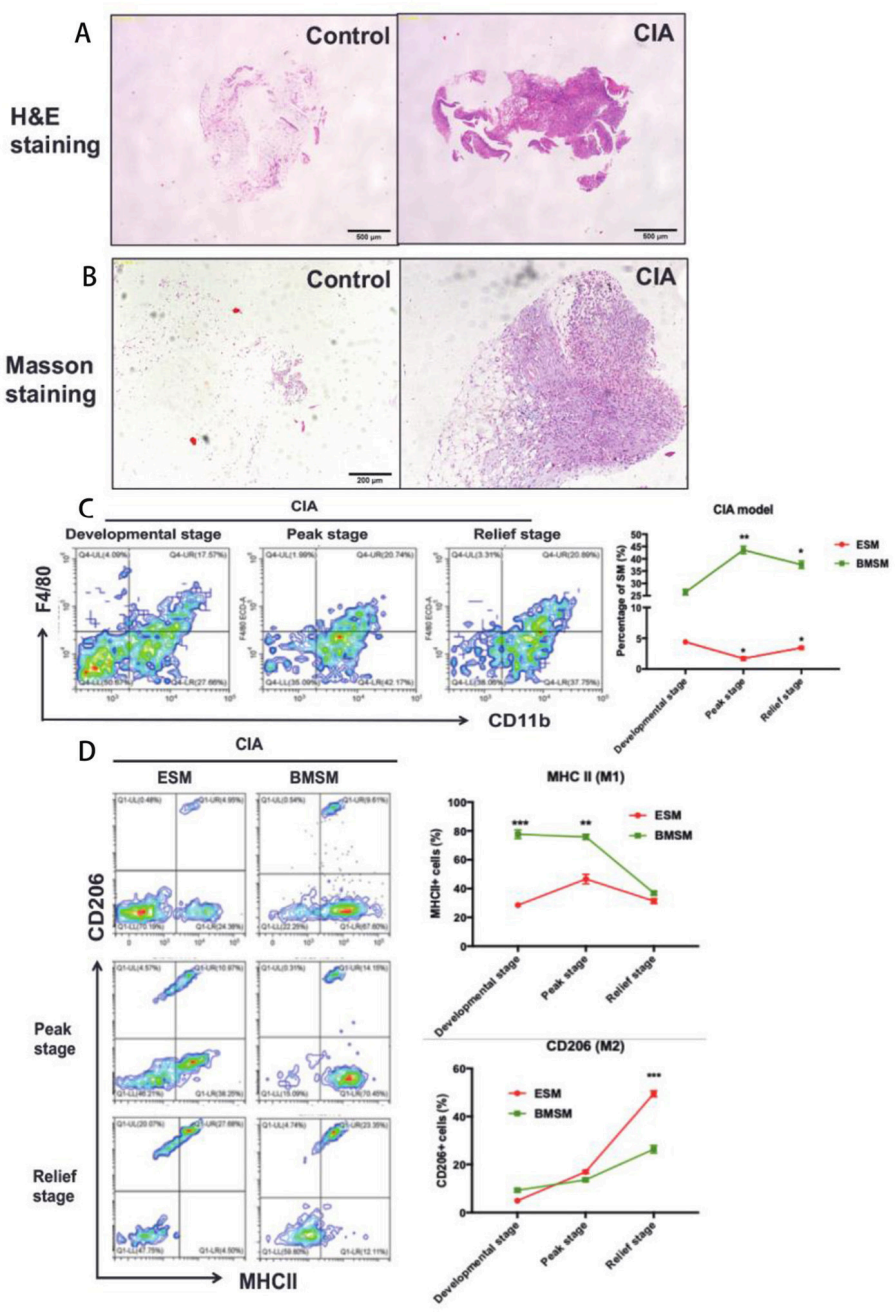
The role of SM from different origins in CIA mice. **(A,B)** H&E staining and Masson staining of synovium and joint of control and CIA mice; **(C)** The dynamic expression pattern of SM in the developmental process of CIA model; **(D)** The expressionfo M1(pro-inflammatory)/M2(anti-inflammatory) markers of ESM and BMSM at different developmental stage of CIA model. Each bar in the figure represents mean ± SEM of triplicates. 5 mice were used for each FACS experiment. *P < 0.05, **P < 0.01, ***P < 0.001.

**Figure 6:**
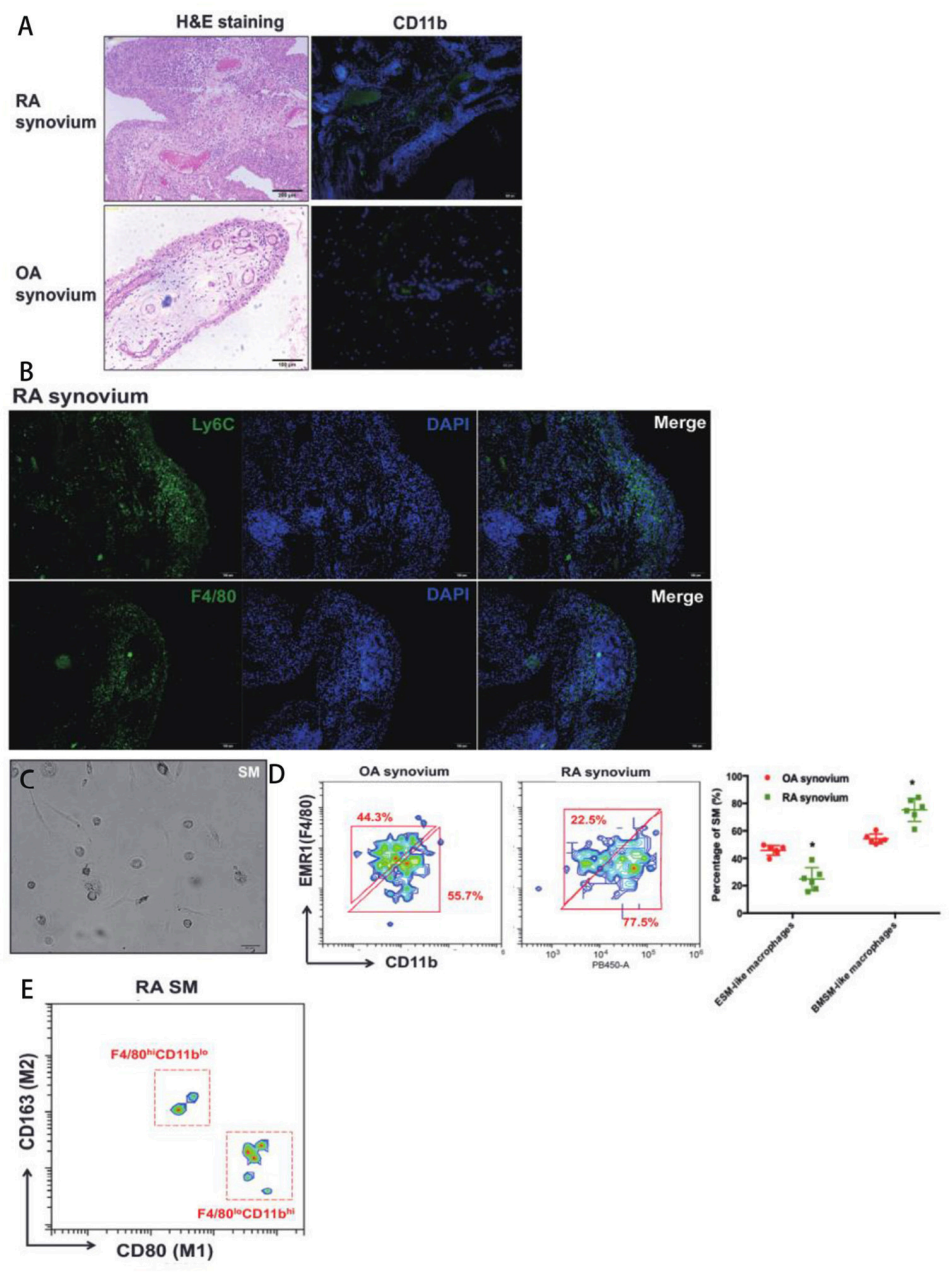
The role of SM from different origins in RA patients' synovium. **(A)** H&E staining and CD11b immunofluorescence of synovium from OA and RA patients; **(B)** EMR1(F4/80) and CD11b immunofluorescence in RA synovium; **(C)** The *in vitro* culture of isolated EMR1(F4/80)^+^CD11b^+^ SM from RA synoivum by using a similar sorting strategy in mice; **(D)** The ratio of ESM and BSM in OA and RA synovium; **(E)** The expression of M1(pro-inflammatory)/M2(anti-inflammatory) markers of ESM and BMSM in RA synovium. Each bar in the figure represents mean ± SEM of six experiments. *P < 0.05.

Some macrophage populations show pro-inflammatory cellular feature under arthritis condition. SM has been proven that play an essential role in Rheumatoid arthritis (RA). However, there is little study to investigate specific role of ESM and BMSM during pathogenesis of RA. Here, a Collagen-induced arthritis (CIA) mice model and replaced synovium from RA patients were used to evaluate the role of ESM and BMSM in arthritis. Firstly, synovium was isolated from joints of CIA mice. Compared to normal control, H&E staining and Masson staining showed that the hyperplasia and hyper-infiltration of immune cells were observed in joint synovium that isolated from CIA mice (Figures 5A,B). Next, we detected the dynamic expression pattern of SM in the developmental process of CIA model (Figure 5C). ESM (F4/80^+^CD11b^−^) gradually decreased from initial developmental stage to peak stage, and then increased at relief stage. On contrary, BMSM (F4/80^−^CD11b^+^) showed an opposite expression pattern. We further analyzed the M1(pro-inflammatory) and M2(anti-inflammatory) percentage of ESM and BMSM at different developmental stage of CIA model (Figure 5D). FACS results demonstrated that the polarization of ESM skewed to M2 while BMSM showed more M1 phenotype during the CIA development. Taken together, these data suggested that ESM is M2-like population that was generally repressed in CIA model; while the number of BMSM population significantly increased in CIA model and showed M1-like pro-inflammatory phenotype. To further prove this point in human, synovium from RA patients joint was used. SM from osteoarthritis (OA) patients were used as control, since the inflammatory features of OA are much less than that of RA (Figure 6A and Supplementary Figure 5). Immunofluorescence results suggested that there are much more CD11b^+^ cells infiltration in RA synovium than that of OA synovium (Figure 6A). In RA synovium, CD11b^+^ cells localized within and around vessel tube and EMR1(the human homology of mouse F4/80)^+^ cells appeared outside vessel (Figure 6B), validating that BMSM recruitment was from circulation and significantly increased in RA synovium. On contrary, EMR1^+^ cells didn't localize near blood vessel, suggesting that ESM wasn't circulation-orient (Figure 6B). EMR1^+^CD11b^+^ RA SM was also isolated from RA and OA synovium by using a similar sorting strategy in mice (Figure 6C). RA SM showed much more BMSM population (EMR1^low^CD11b^high^) and less ESM population (EMR1^high^CD11b^low^), mimicking the results from CIA mice (Figure 6D). Similar to CIA mice, BMSM population (EMR1^low^CD11b^high^) and ESM population (EMR1^high^CD11b^low^) from RA synovium demonstrated M1 and M2 polarization, respectively (Figure 6E). In summary, these results suggested that BMSM is more like a group of pro-inflammatory SM, which significantly increased in both CIA mice and RA patients, while ESM is functional as an anti-inflammatory role during the same conditions.

## Discussion

Recently, the traditional idea that tissue macrophages are exclusively derived from circulating monocytes has been challenged. A series of paper suggested that tissue macrophages stem from both embryonic precursors except circulating monocytes (5, 13, 15). In the current study, we found that SM also has two origins: ESM seeded at joint symnovium at prenatal stage and the classic BMSM also infiltrated in synovium at perinatal stage. This two distinct SM populations gradually mixed together with development after birth. This transition is a potential key step for physiological homeostasis of joint synovium. In addition, we found that ESM are radio-resistant in chimera model. This is a surprising finding because macrophages in most peripheral tissue are, at least partially, radio-sensitive, such as bone marrow macrophages (16), colonic macrophages (1) and langerhans cells (7). This feature shows the unique character of SM. Therefore, the difference between SM and other resident peripheral tissues should be compared in the future.

The cellular features of these two SM populations are also quite different, including proliferation, phagocytosis and expression of pro-inflammatory and anti-inflammatory genes. Of interest, ESM showed considerable proliferative ability during the prenatal and neonatal periods (17, 18), suggesting that the cellular phenotype might change with the development. To further elucidate this, embryonic pulse-chase fate mapping system will be used to compare ESM in prenatal, neonatal and adult stages.

Due to the therapeutic potential of changing macrophage phenotype in RA, the polarization of macrophages plays an essential role in many pathological conditions. ESM and BMSM from CIA mice show specific M1 and M2 polarization trends, implying that the complexity of SM is also high. Interestingly, compared to CIA mice, we got a similar result of SM polarization from synovium from RA patients, suggesting that altering macrophage polarization in autoimmune inflammation is indeed a potential treatment that definitely warrants further investigation.

## Ethics Statement

This study was carried out in accordance with the recommendations of No. 20160094, biomedical ethics committee of Anhui Medical University with written informed consent from all subjects. All subjects gave written informed consent in accordance with the Declaration of Helsinki. The protocol was approved by the biomedical ethics committee of Anhui Medical University.

This study was carried out in accordance with the recommendations of No. LLSC20160121, experimental animals ethics committee of Anhui Medical University. The protocol was approved by the experimental animals ethics committee of Anhui Medical University.

## Author Contributions

JT, YG, WH, PZ, XW, YF, XC, and SL performed the experiments and drafted the manuscript. WW revised the manuscript.

### Conflict of Interest Statement

The authors declare that the research was conducted in the absence of any commercial or financial relationships that could be construed as a potential conflict of interest.

## Supplementary Material

Supplementary Figure 1 The preparation and gating process for ESM and BMSM sorting.

Supplementary Figure 2 The morphology of mouse synovium at E15.5 and P7 stage.

Supplementary Figure 3 The expression of pan-macrophage CD68 in F4/80-CD11b+ BMSM during perinatal stage.

Supplementary Figure 4 The expression pattern of ESM, BMSM, and F4/80^+^CD11b^+^ SM at postnatal stage and the total numbers of SM during mice development.

Supplementary Figure 5 Safranin-O staining of OA and RA synovium.
