# Supplementary material for: Ontogeny of Synovial Macrophages and the Roles of Synovial Macrophages From Different Origins in Arthritis
Source: Front Immunol. 2019 May 24;10:1146. doi: 10.3389/fimmu.2019.01146 (PMC6558408; doi:10.3389/fimmu.2019.01146)

Supplementary figure 1

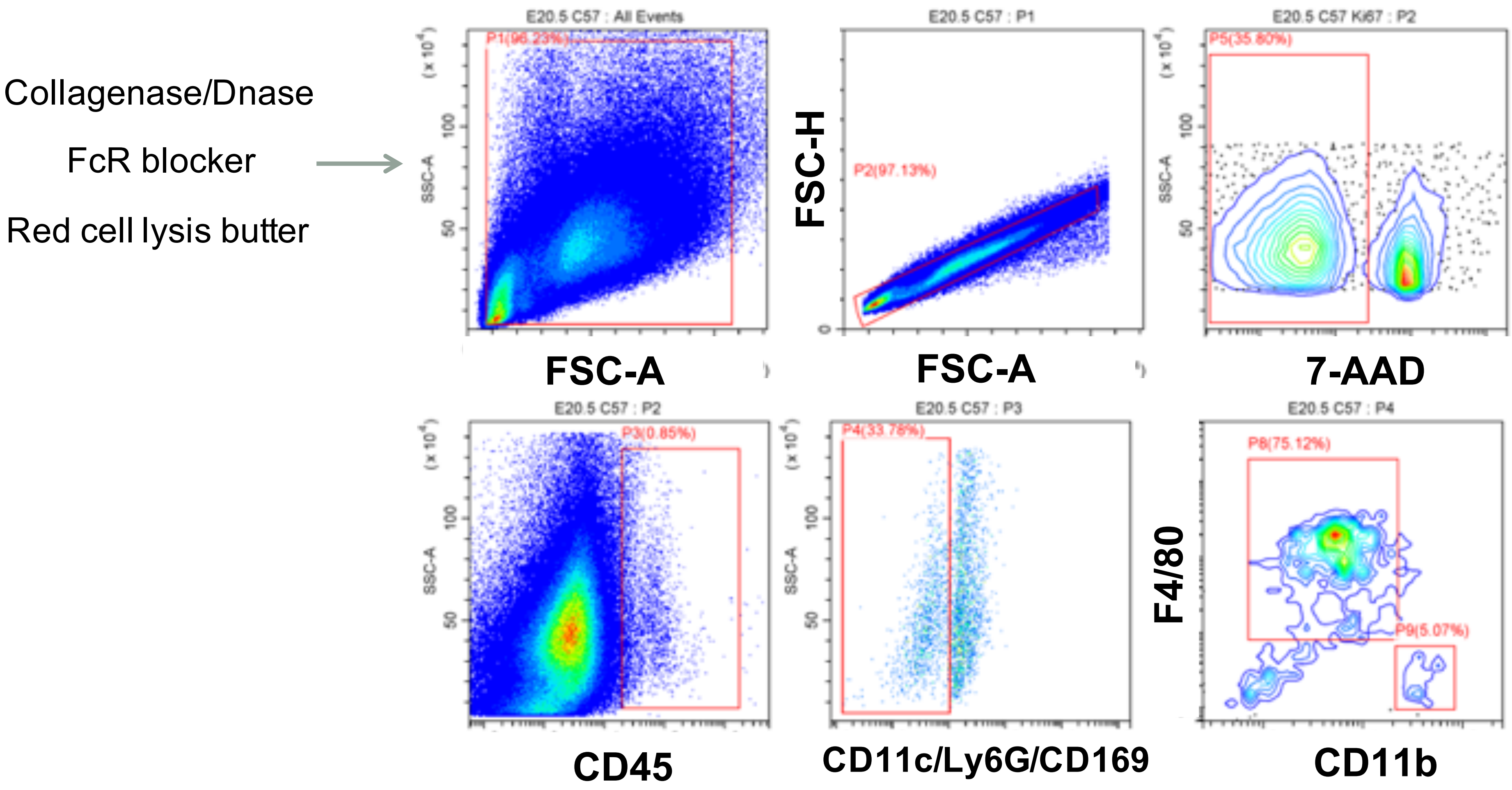

Supplement: Supplementary Figure 1 — The preparation and gating process for ESM and BMSM sorting. [file Data_Sheet_1.pdf]
