# Supplementary figures and images for: Ontogeny of Synovial Macrophages and the Roles of Synovial Macrophages From Different Origins in Arthritis
Source: Front Immunol. 2019 May 24;10:1146. doi: 10.3389/fimmu.2019.01146 (PMC6558408; doi:10.3389/fimmu.2019.01146)

Supplementary figure 2

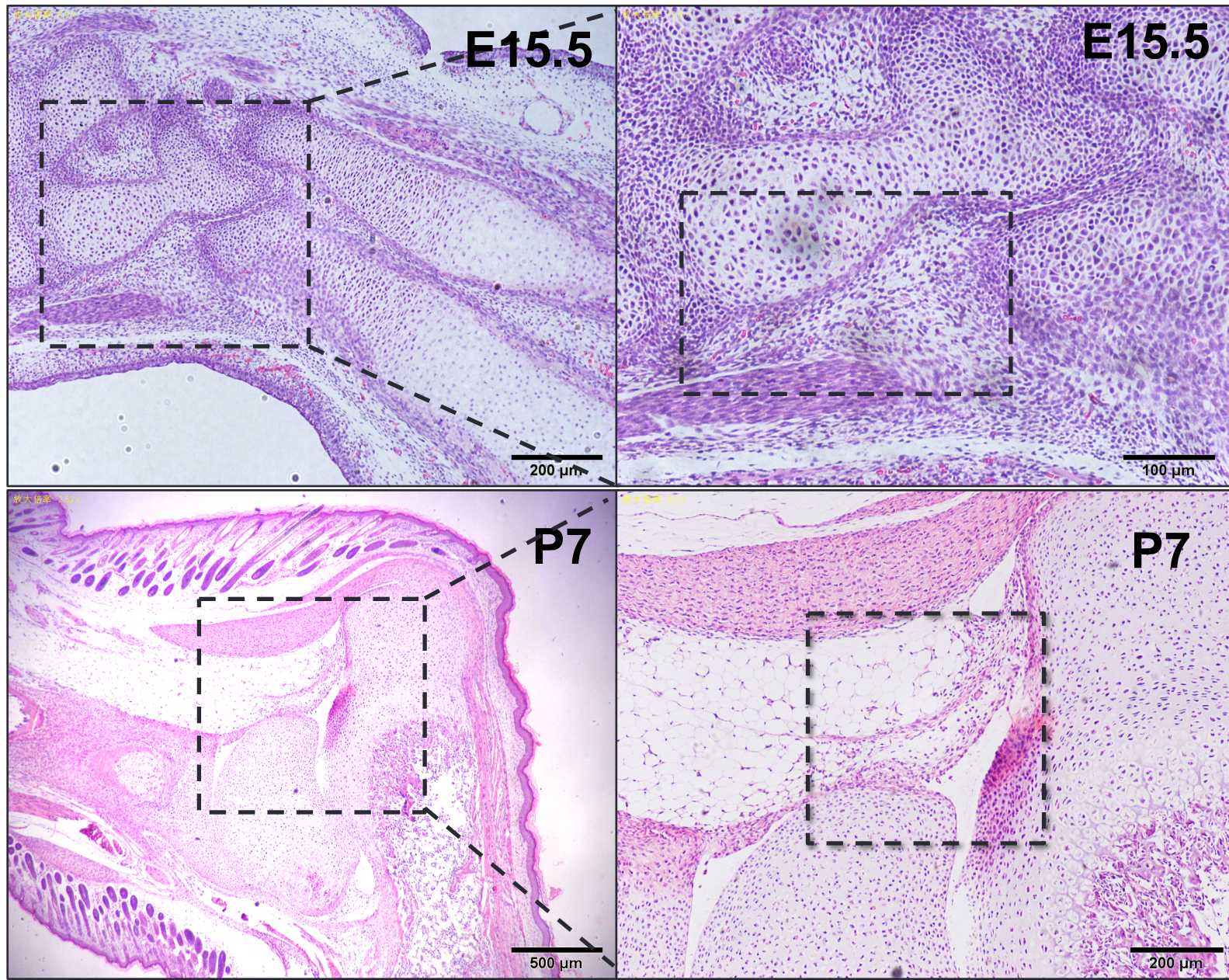

Supplement: Supplementary Figure 2 — The morphology of mouse synovium at E15.5 and P7 stage. [file Data_Sheet_2.pdf]

# Supplementary figure 3

## CD11b+F4/80- BMSM

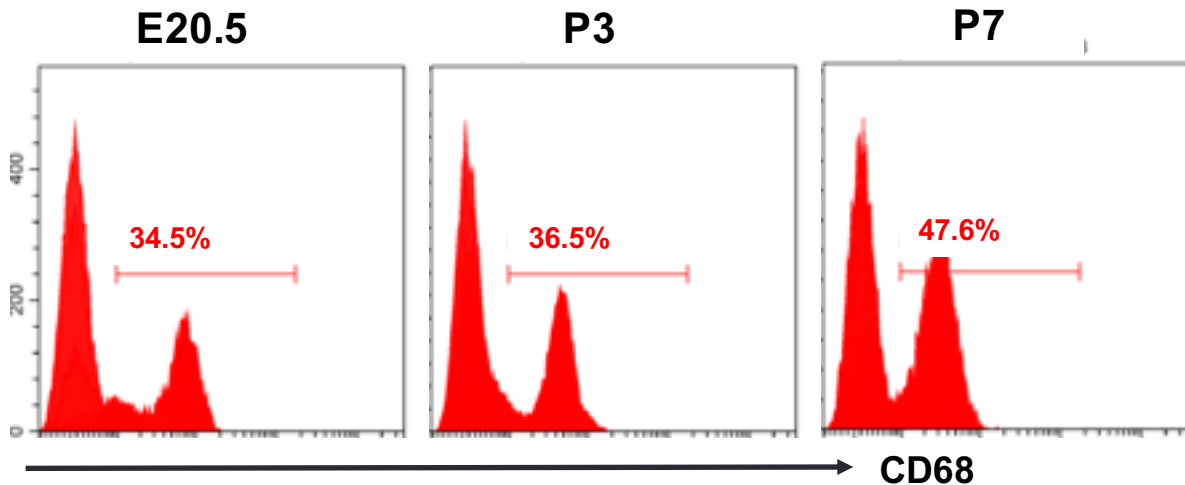

Supplement: Supplementary Figure 3 — The expression of pan-macrophage CD68 in F4/80-CD11b+ BMSM during perinatal stage. [file Data_Sheet_3.pdf]

## Supplementary figure 4

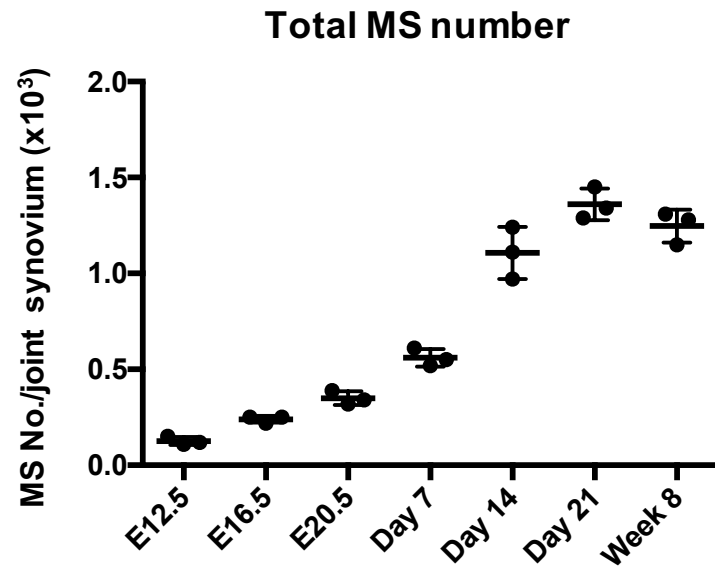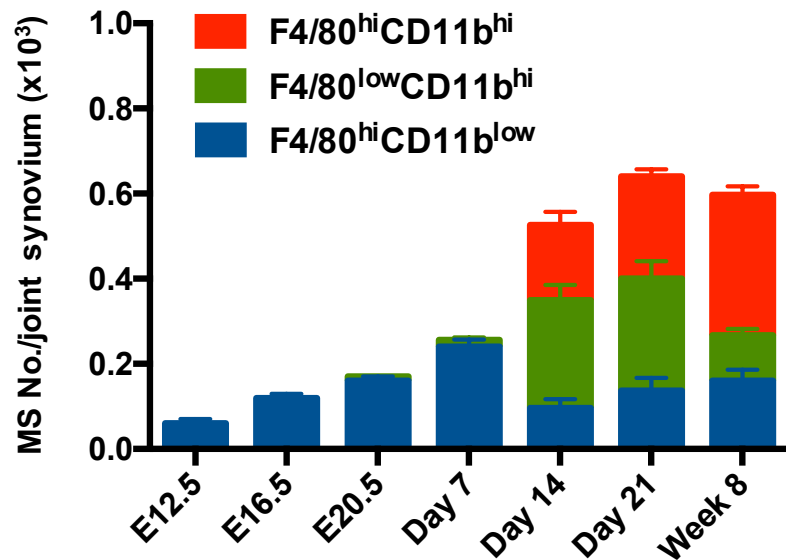

Supplement: Supplementary Figure 4 — The expression pattern of ESM, BMSM, and F4/80+CD11b+ SM at postnatal stage and the total numbers of SM during mice development. [file Data_Sheet_4.pdf]

# Supplementary figure 5

**OA synovium**

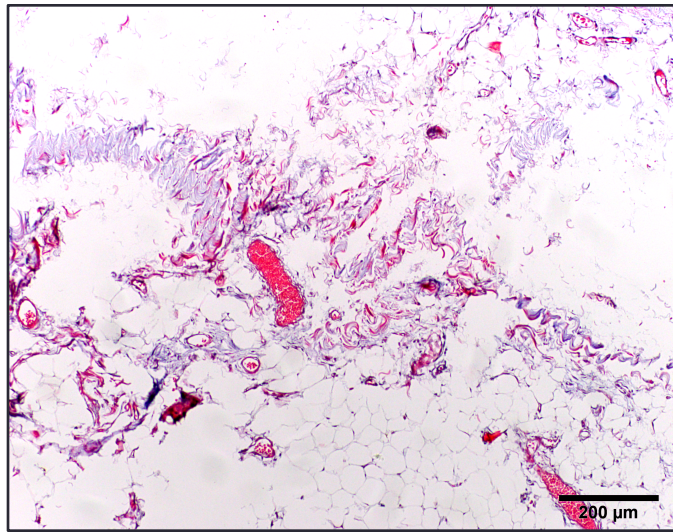

**RA synovium**

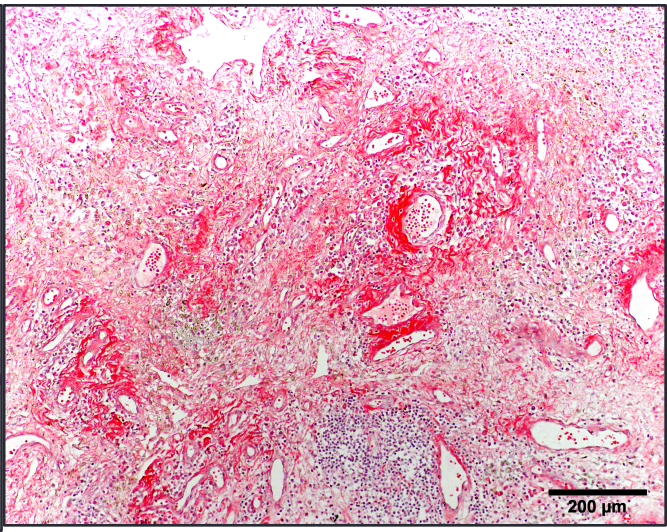

Supplement: Supplementary Figure 5 — Safranin-O staining of OA and RA synovium. [file Data_Sheet_5.pdf]
